# Methanol Gas-Sensing Properties of SWCNT-MIP Composites

**DOI:** 10.1186/s11671-016-1675-3

**Published:** 2016-11-25

**Authors:** Jin Zhang, Qin Zhu, Yumin Zhang, Zhongqi Zhu, Qingju Liu

**Affiliations:** 1 School of Materials Science and Engineering, Yunnan Key Laboratory of Micro/Nano Materials and Technology, Yunnan University, Kunming, 650091 People’s Republic of China; 2 The State Key Laboratory of Advanced Technologies for Comprehensive Utilization of Platinum Metals, Sino-Platinum Metals Co., Ltd., Kunming, 650106 People’s Republic of China

**Keywords:** SWCNT-MIP composites, Gas-sensing properties, Methanol gas

## Abstract

**Abstract:**

The single-walled carbon nanotube (SWCNT)-molecularly imprinted powder (MIP) composites in this paper were prepared by mixing SWCNTs with MIPs. The structure and micrograph of the as-prepared SWCNTs-MIPs samples were characterized by XRD and TEM. The gas-sensing properties were tested through indirect-heating sensors based on SWCNT-MIP composites fabricating on an alumina tube with Au electrodes and Pt wires. The results showed that the structure of SWCNTs-MIPs is of orthogonal perovskite and the average particle size of the SWCNTs-MIPs was in the range of 10–30 nm. SWCNTs-MIPs exhibit good methanol gas-sensitive properties. At 90 °C, the response to 1 ppm methanol is 19.7, and the response to the interferent is lower than 5 to the other interferent gases (ethanol, formaldehyde, toluene, acetone, ammonia, and gasoline). The response time and recovery time are 50 and 58 s, respectively.

**Graphical Abstract:**

The as prepared SWCNTs-MIPs possesses good selectivity and high response to low concentrationmethanol
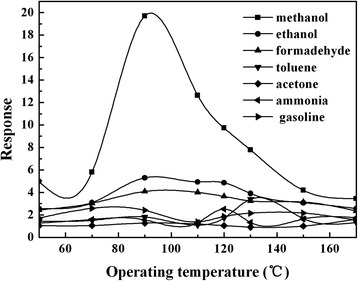

## Background

Perovskite oxides with ABO_3_ structure (A, rare earth; B, transition metal) have been shown to have excellent gas-sensing properties [1–5]. For some p-type ABO_3_ semiconductors that are prepared in air condition, their resistance will be decreased when they adsorb oxidizing gas while their resistance will be increased when they adsorb reducing gas. This special property could be employed in the application of gas sensor to detect different gas in the atmosphere. Zhang et al. [6] investigated the formaldehyde-sensing properties of Ag-LaFeO_3_ system and found that Ag-LaFeO_3_ sensors exhibited good performance to formaldehyde gas. Giang et al. [7] reported that the mixed potential sensor based on Pt/YSZ/SmFeO_3_ had very high sensitivity to NO_2_ at the operating temperature from 300 to 500 °C. Chen et al. [8] investigated the SmFe_1 − x_Ni_x_O_3_ sensors and showed that these sensors exhibited good performance to ethanol gas. It was showed that single-walled carbon nanotube (SWCNT) incorporation of Ag-LaFeO_3_ system lowered the operating temperature effectively and increased the sensing response to formaldehyde gas [9]. Doroftei et al. [10] designed a La–Pb–Fe–O perovskite gas sensor of methanol, and the highest response of the sensor to 400 ppm methanol gas is 146.6 at 230 °C.

Molecular imprinting is a technique for the introduction of selective recognition sites into highly cross-linked polymeric matrices, in which the functionalized monomers are introduced into the polymer network via direct-template-assemble [11, 12]. Generally, polymerization acts as print molecule or template and forms the complex with the constituent monomers. When the templates are removed, the cavities that keep specific recognition sites are left in the polymeric structure. Today, molecular imprinting has been successfully used in the fields of enzyme-mimicking catalysts, chromatographic separation, biosensors, and chemical sensors [13–15]. However, most of the analyte molecules are organic macromolecule, such as tryptophan, caffeine, glutamic acid, and bisphenol, and there is no report on using the molecular imprinting technique in semiconductor oxides to recognize small organic molecules in addition to our previous work [16, 17]. SWCNTs have been the most actively studied materials in recent years due to their special nanostructure, large specific surface area, and excellent electrical properties [18]. As reported, SWCNTs are very sensitive to the surrounding environment. The presence of O_2_, NO_2_, and NH_3_ gases and many other molecules can either donate or accept electrons, resulting in an alteration of the overall conductivity [19, 20]. SWCNTs become the ideal gas-sensitive materials because of their excellent properties. Recently, the combination of semiconductor metal oxide with SWCNTs has been explored to modify semiconductor metal oxide gas sensors, and many interesting findings have been obtained [21, 22].

In our previous work [17], a new methanol gas sensor was prepared by using molecularly imprinted powders (MIPs). The sensor has many good characteristics, such as selectivity, stability, and response-recovery characteristics, but its optimal operating temperature is still unsatisfactory (around 130 °C). In order to reduce the optimal operating temperature, MIPs were modified with SWCNTs. The results show that SWCNT-MIP composites exhibited excellent gas-sensing properties for methanol gas and the optimal operating temperature has been reduced to 90 °C.

## Methods

### Preparation of MIPs

All the chemical reagents used in the present work were obtained from commercial sources as guaranteed-grade reagents and used without further purification.

LaFeO_3_ of perovskite structure oxides was prepared by a sol-gel method. The detailed process was as follows: Fe(NO_3_)_3_·9H_2_O, La(NO_3_)_3_·6H_2_O, and citrate with mole ratio (Fe^3 +^)/(La^3 +^)/citrate = 1:1:1 were first dissolved in distilled water, and subsequently, polyethylene glycol (PEG) was added. Finally, the mixed solution was stirred at 80 °C for 8 h to get LaFeO_3_ sol. The functional monomer of methylacrylic acid (MAA) was mixed with methanol (which acts as a template molecule) in a reaction vial. Then, the cross-linking agent of LaFeO_3_ sol was added into the MAA solution with various molar ratios (*x* = MAA:LaFeO_3_ = 1:10, 4:10, 6:10, 8:10, 10:10), the radical initiator of azodiisobutyronitrile (AIBN) was added to the mixture, and the mixture was stirred for polymerization at 50 °C for 12 h under the protection of N_2_. After this, the resulting polymer was ground and dried at 80 °C to remove the template molecule completely.

### Preparation of SWCNT-MIP Composites

The MIPs (*x* = 6:10) were modified with SWCNTs (*w* = SWCNTs:MIPs = 0.25, 0.50, 0.75, 1.00, 1.25, and 1.50% weight ratio). SWCNTs and MIPs were treated by ultrasonic dispersion in the distilled water for 30 min. Subsequently, the mixture was put in a microwave chemical device for 2 h and then dried. The composites were finally obtained.

### Fabrication of Gas Sensors

In the present work, the gas sensors were fabricated in the following process: the SWCNT-MIP composites were mixed with deionized water to form paste, and then coated onto the outside of an alumina tube with Au electrodes and Pt wires. A Ni-Cr alloy wire crossing the alumina tube was used as a resistor which ensured both substrate heating and temperature control. All the gas sensors were aged at operating temperature 150 °C for 170 h in air in order to improve their stability and repeatability. The gas response was defined as the ratio of the electrical resistance in gas (Rg) to that in air (Ra) [6].

### Characterization

X-ray diffraction (XRD, D/max23) with Cu Kα radiation (*λ* = 1.54056 Ǻ) and transmission electron microscope (TEM, JEM-2100) were used for the phase identification and morphology of the samples. The infrared spectra were identified by FTS-40 infrared spectrometer, and the sample was scanned from 4000 to 400 cm^−1^ with a KBr pellet method.

## Results and Discussion

Gas-sensing properties of the MIPs with *x* = 6:10 (labeled “Sample-A” in the following text) and the SWCNT-MIP composite with *w* = 1.00% (labeled “Sample-B” in the following text) are better than those with *x* = 1:10, 4:10, 8:10, and 10:10 and those with *w* = 0.25, 0.50, 0.75 1.25, and 1.50%, respectively. So in this paper, we mainly discuss Sample-A and Sample-B.

### Structure and Morphology of Gas-Sensing Materials

**Fig. 1:**
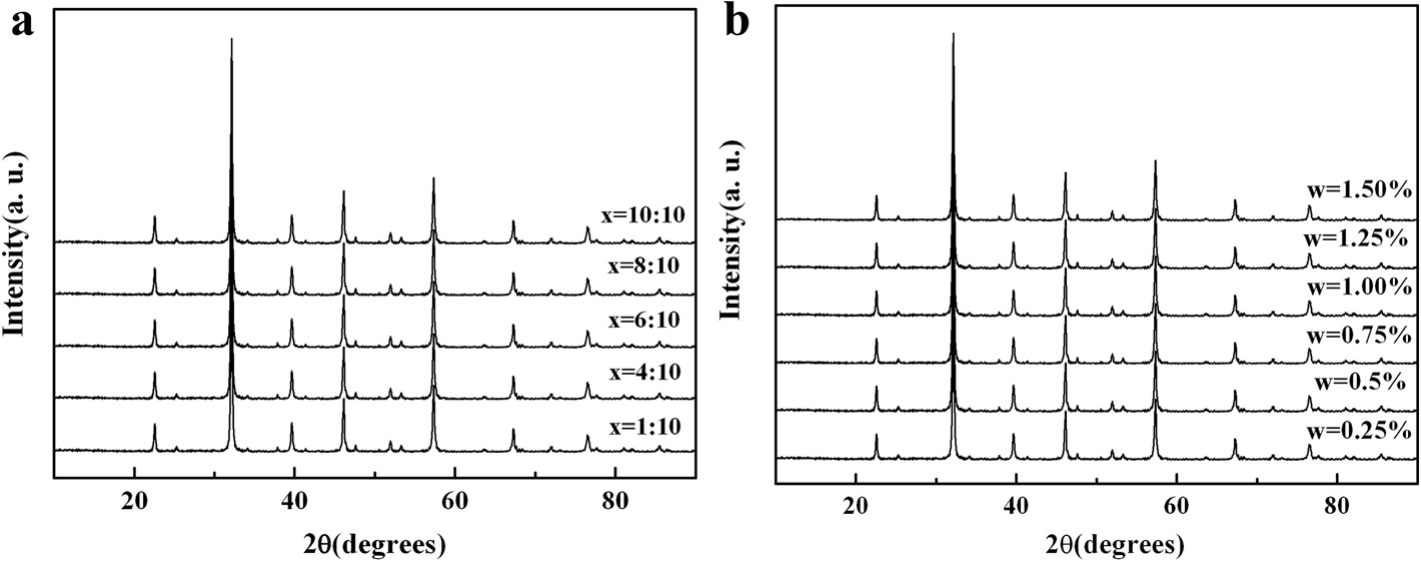
XRD patterns of **a** MIPs (1:10 ≤ *x* ≤ 10:10) and **b** SWCNT-MIP composites (0.25% ≤ *w* ≤ 1.50%)

The XRD patterns of MIPs and SWCNT-MIP composites with different proportions are displayed in Fig. 1. The results indicate that all the peaks of the pattern of the MIPs and SWCNT-MIP composites could belong to the orthogonal perovskite structure of LaFeO_3_. No other impurity peaks are observed in the figures. This is because the amount of functional monomer and SWCNTs is so small that they cannot be detected. The AIBN and the template molecules are removed from MIPs in the process of drying.

**Fig. 2:**
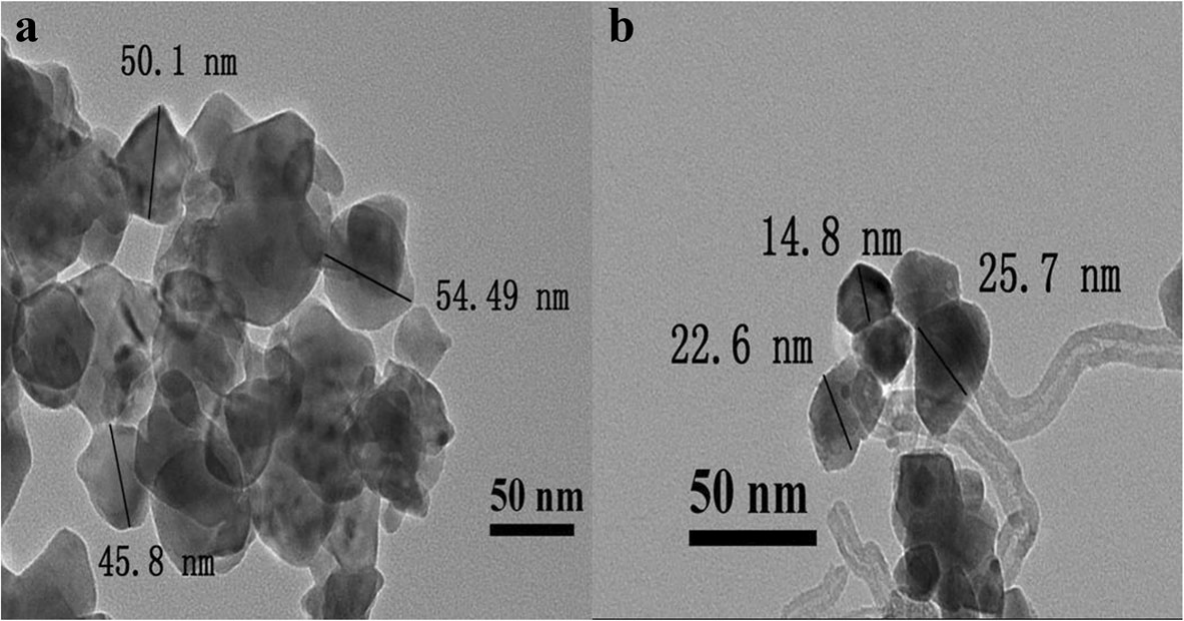
TEM images of the materials of **a** Sample-A and **b** Sample-B

Figure 2 shows the TEM images of Sample-A and Sample-B. From the image of Fig. 2a, we can see the particles of Sample-A are generally irregular and agglomerated. The particle size is in the range of 40–60 nm. In Fig. 2b, Sample-B are many spherical particles which are uniform in size and dispersed on the surface of SWCNTs. The particle size is in the range of 10–30 nm. It can be seen that the particle sizes of Sample-B (Fig. 2b) are smaller than that of Sample-A (Fig. 2a). The smaller and better dispersed particles result in larger specific surface area, which are very benefit to the gas-sensing reactions between the materials surface and the gases.

### IR Analysis

**Fig. 3:**
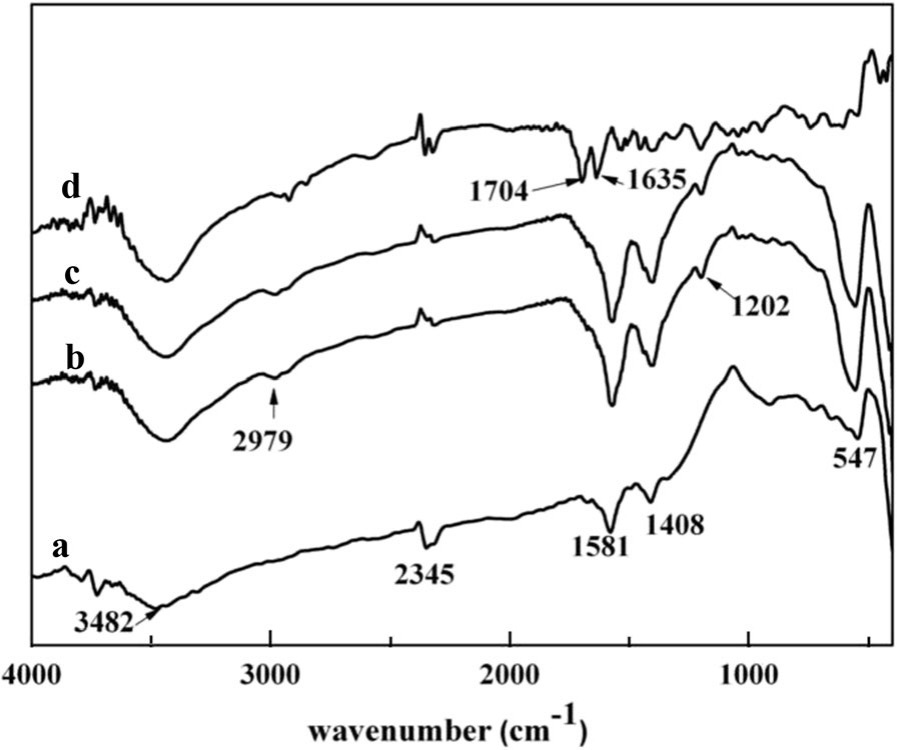
FT-IR spectroscopy of (*a*) LaFeO_3_, (*b*) Sample-A, (*c*) Sample-B, and (*d*) MAA

Figure 3 shows the FT-IR spectroscopy of LaFeO_3_ (a), Sample-A (b), Sample-B (c), MAA (d) in the range of 400–4000 cm^−1^. In the case of LaFeO_3_, the peak occurring at 3482 cm^−1^ is attributed to the stretching vibration of O–H of H_2_O in air, and the other peaks at 547 and 2345 cm^−1^ are corresponding to Fe–O vibrations and the gas phase carbon dioxide vibrations, respectively. The peaks at 1408 and 1581 cm^−1^ represent the La–O vibrations [23]. In the case of Sample-A and Sample-B, new peaks appearing at 1202 and 2979 cm^−1^ compared with LaFeO_3_ are attributed to the stretching vibration of C–O and O–H in carboxylic acid, respectively. Compared with Sample-A and Sample-B, the relatively strong peaks of C=O (1635 cm^−1^) stretching vibration in carboxylate anion and C=O (1704 cm^−1^) stretching vibration in carboxylic acid are disappearing, indicating the presence of interaction that is ascribed to the coordination between carbonyl groups in MAA and La in LaFeO_3_ [24]. Moreover, the IR curves of Sample-A and Sample-B show almost identical wave numbers and positions for the main bands. In these curves, the related vibration peaks of SWCNTs are hardly found. This is because the intensity of the transmission light to the SWCNTs is very low, and the corresponding reflective or scattering light has to transfer the MIPs [25].

### Gas-Sensing Performance of the Sensors

**Fig. 4:**
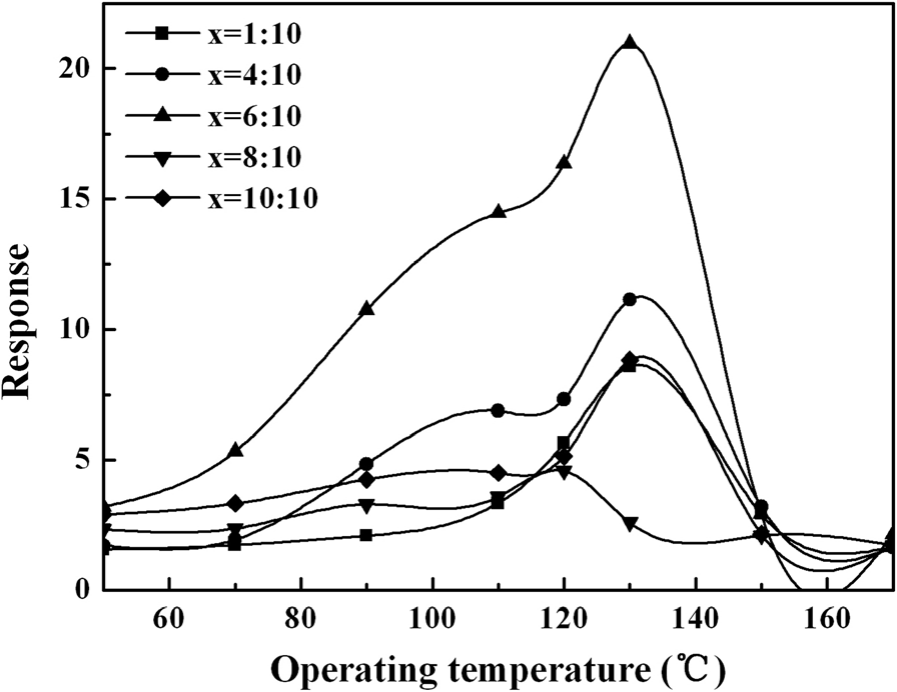
Response-operating temperature curves for 1 ppm methanol gas of MIPs (1:10 ≤ *x* ≤ 10:10) sensors

Figure 4 shows the relationship between the response and operating temperatures of the MIPs (1:10 ≤ *x* ≤ 10:10) sensors to 1 ppm methanol gas. In the lower operating temperature range (50~150 °C), the sensor with *x* = 6:10 exhibits higher response for methanol gas. On the other hand, sensors with lower ration of MAA (1:10 ≤ *x* ≤ 4:10) exhibit poor gas-sensing properties because the interaction between the template molecule (methanol) and functional monomer (MAA) is relatively weak and specific recognition sites in the MIPs for the methanol are reduced. However, if the ration of MMA is creasing, MMA molecules will form polymers via auto-polymerization, which also lead to poor gas-sensing performance. Thus, the optimal ratio of MMA is *x* = 6:10 (i.e., Sample-A) for MIPs in the present work.

**Fig. 5:**
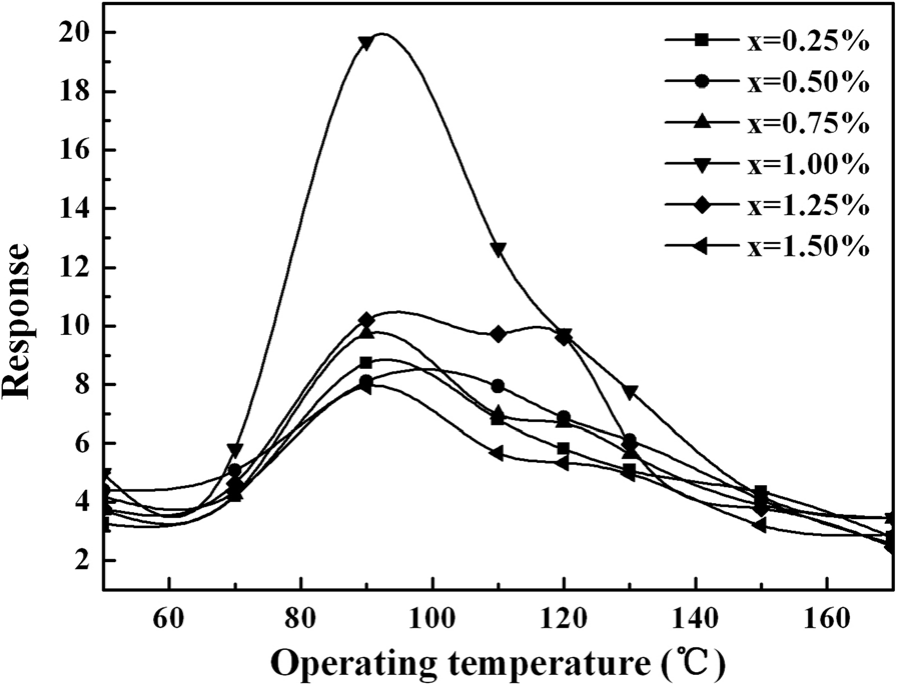
Response-operating temperature curves for 1 ppm methanol gas of SWCNTs-MIPs composites (0.25% ≤ *w* ≤ 1.50%) sensors

Figure 5 shows the operating temperature dependence of the gas response of SWCNT-MIP composites with different ratios of SWCNTs (*w* = SWCNTs:MIPs = 0.25, 0.50, 0.75, 1.00, 1.25, and 1.50% weight ratio) to 1 ppm methanol gas. It can be seen that the sensor with *w* = 1.00% (Sample-B) exhibit higher response to methanol gas at the operating temperature between 50 and 150 °C. If the amount of SWCNTs is lower (*w* = 0.25, 0.50, 0.75%), the particles of the MIPs cannot be separated effectively and the size of particles cannot be refined, which result in bad gas-sensing properties. On the contrary, if the amount of SWCNTs is higher (*x* = 1.25, 1.50%), most SWCNTs are aggregated or coated on the surface of the MIPs, which lead to particle size increase and poor gas-sensing performance. Taking the conception of sensing performance and cost conservation into consideration, for SWCNT-MIP composites, *w* = 1.00% (Sample-B) is the optimal ratio.

**Fig. 6:**
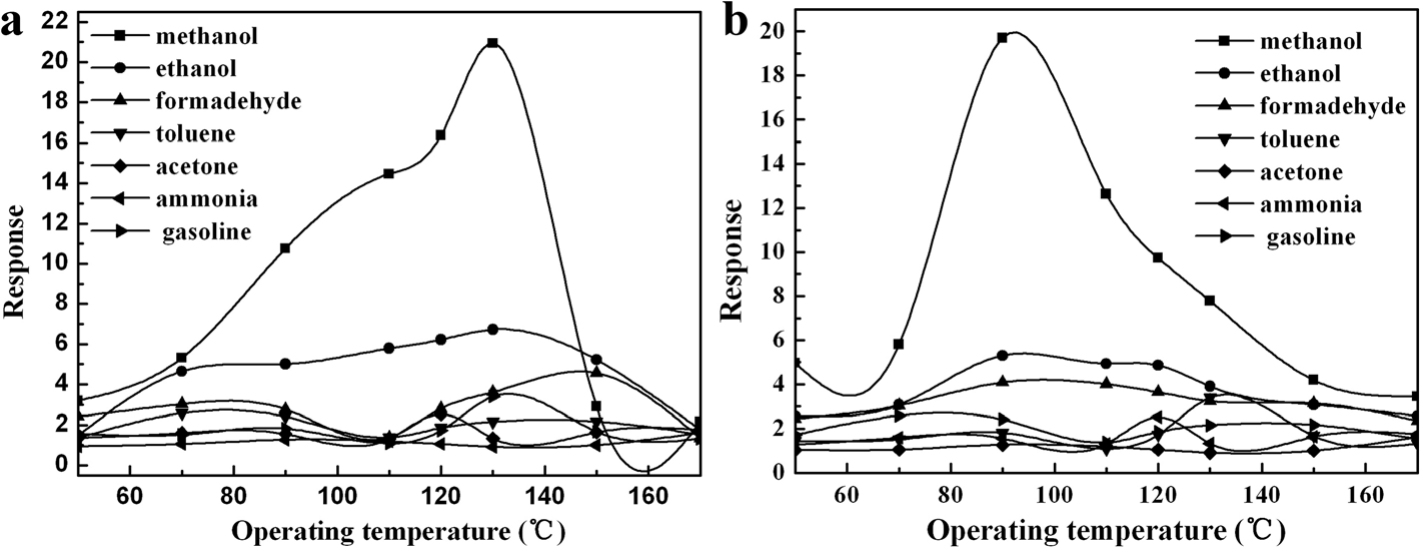
Response-operating temperature curves for 1 ppm different tested gases of the **a** sensor based on Sample-A and **b** sensor based on Sample-B

Figure 6 shows the operating temperature dependence of the gas response of Sample-A and Sample-B to 1 ppm methanol, ethanol, formaldehyde, toluene, acetone, ammonia, and gasoline, respectively. It can be found that the Sample-A and Sample-B sensors behave well in selectivity for methanol gas. In Fig. 5a, the best response to 1 ppm methanol gas of Sample-A sensor is 21 at the operating temperature of 130 °C, while it shows a lower response (≤7) to the other test gases. In Fig. 5b, the best response to 1 ppm methanol gas of Sample-B sensor is 19.7 at 90 °C, and to the other test gases, the highest response is lower than 5. The results indicate that the optimal operating temperature (90 °C) of the sensor based on Sample-B is lower than that of the sensor based on Sample-A (130 °C), and the selectivity to methanol is further increased. The reason for the reduction of the optimal operating temperature of the Sample-B sensor may be ascribed to (1) the particle size of Sample-B is smaller than that of Sample-A after modified with SWCNTs, the particles are dispersed (in Fig. 2b), and thus, the specific surface area of Sample-B is increased, which can provide more active sites for adsorption of methanol gas and increase the selectivity and (2) SWCNTs can transport the electrons easily [26, 27], which can reduce the resistance of the sensor. With lower resistance, the sensor can operate at lower temperature. In summary, compared to Sample-A, Sample-B sensor exhibit lower operating temperature and better selectivity while it maintains a high response. So, we mainly discuss Sample-B sensor in the following text.

**Fig. 7:**
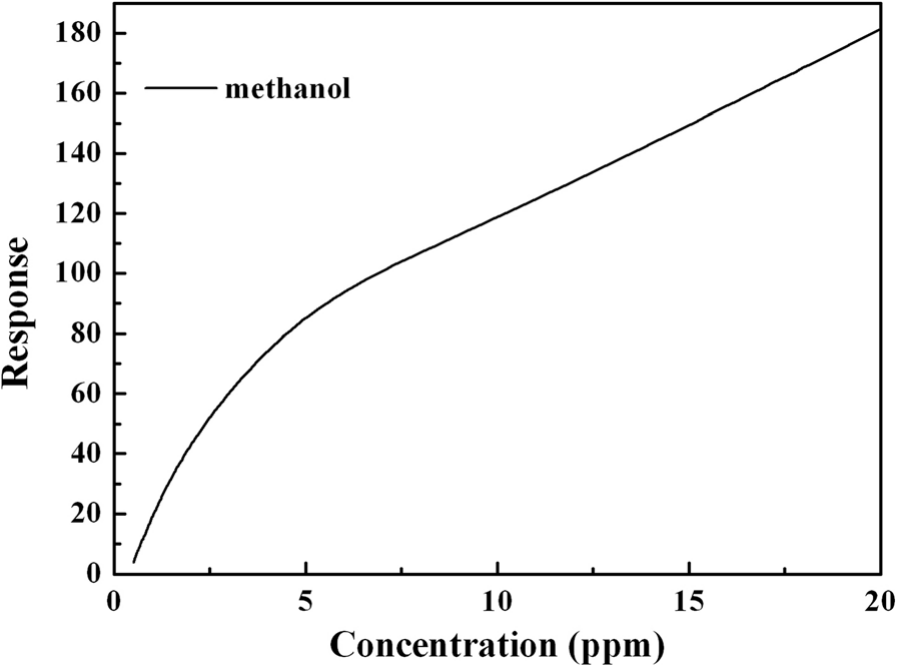
The relationship of response of Sample-B and concentration of methanol gas at 90 °C

The correlation between methanol gas concentration and Sample-B gas response at 90 °C is shown in Fig. 7. It can be seen that the gas response was increased linearly by the enhancement of the methanol gas concentration in the range of 0.5–20 ppm. So the Sample-B sensors fabricated can be used as a continuous real-time monitoring at lower concentration of methanol gas.

**Fig. 8:**
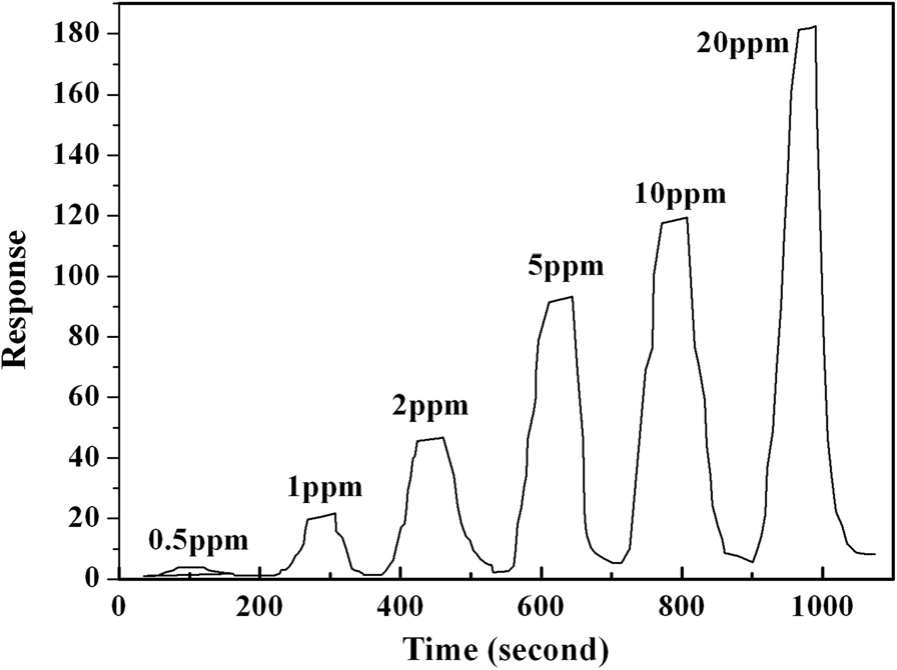
Response-recovery time curve of the sensor based on Sample-B operated at 90 °C

Figure 8 shows the dynamic response change of Sample-B with different methanol gas concentration (0.5–20 ppm) at the operating temperature of 90 °C. The response time and recovery time are 50 and 58 s, respectively. From Fig. 8, Sample-B composites also presented good repetition property. On and off cycles could be repeated several times, and any major changes in the response were observed. All these advantages endow it with the potential to be practical detectors.

The gas-sensing mechanism of MIPs sensors has already been explained in our previous work [17]. After the MIPs modified with SWCNTs, the gas-sensing mechanism is similar to that of MIPs, but the sensing properties of the MIPs could be enhanced by modifying with SWCNTs. (1) The optimal operating temperature of MIPs which modified with SWCNTs reduce effectively from 130 to 90 °C, and (2) the selectivity of SWCNT-MIP composites could be enhanced. The reasons are as follows. (1) SWCNTs can transport the electrons easily, which can reduce the resistance of the sensor. With lower resistance, the sensor can operate at a lower temperature. Thus, modifying with SWCNTs can reduce the operating temperature effectively. (2) The particle size of the MIPs is smaller by adding SWCNTs, so the specific surface area of the SWCNT-MIP composite is increased. Hence, there are much more methanol gas-adsorbing vacancies on the surface of the sensor, which lead to effective adsorption of methanol gas and result in enhancement of the selectivity.

## Conclusions

Perovskite-type MIPs (Sample-A, MAA:LaFeO_3_ = 6:10) and SWCNT-MIP composite (Sample-B, SWCNTs:MIPs = 1.00%) have been prepared. The average particles size of MIPs (MAA:LaFeO_3_ = 6:10) and SWCNT-MIP composite (SWCNTs:MIPs = 1.00%) are about 50 and 20 nm, respectively. The MIP (MAA:LaFeO_3_ = 6:10) and SWCNT-MIP composite (SWCNTs:MIPs = 1.00%) sensors showed high gas sensing to methanol. Compare to MIP sensor, the SWCNT-MIP sensor has lower optimal operating temperature (90 °C) and high selectivity (to 1 ppm methanol, the response is 19.7 at the operating temperature of 90 °C, and to the other test gases, the responses are all lower than 5). These results indicate that the methanol gas-sensing properties of the sensor based on the MIPs can be improved by modifying with SWCNTs, and the SWCNTs modified MIPs is a feasible way for improving the gas-sensing properties of the MIP-based sensors.

## Highlights

The methanol gas-sensing properties of the SWCNT-MIP sample (SWCNTs:MIPs = 1.00%) is the best.

The response of SWCNT-MIP sample (SWCNTs:MIPs=1.00%) to 1.0 ppm methanol gas is 19.7 at 90 °C and lower than 5.0 to the other test gases.
